# Inter- and intraspecific responses of coral colonies to thermal anomalies on Palmyra Atoll, central Pacific

**DOI:** 10.1371/journal.pone.0312409

**Published:** 2024-11-25

**Authors:** Adi Khen, Michael D. Fox, Maggie D. Johnson, Christopher B. Wall, Jennifer E. Smith

**Affiliations:** 1 Center for Marine Biodiversity and Conservation, Scripps Institution of Oceanography, University of California, San Diego, La Jolla, California, United States of America; 2 Red Sea Research Center, King Abdullah University of Science and Technology, Thuwal, Saudi Arabia; 3 Department of Ecology, Behavior, and Evolution, University of California, San Diego, La Jolla, California, United States of America; 4 Department of Earth Sciences, University of Hawai‘i at Mānoa, Honolulu, Hawaii, United States of America; MARE – Marine and Environmental Sciences Centre, PORTUGAL

## Abstract

Long-term monitoring of individual coral colonies is important for understanding variability between and within species over time in the context of thermal stress. Here, we analyze an 11-year time series of permanent benthic photoquadrats taken on Palmyra Atoll, central Pacific, from 2009 to 2019 to track the growth (i.e., increase in live planar area), pigmentation or lack thereof (“discoloration”), partial or whole-colony mortality, survival, and regrowth of 314 individual coral colonies of nine focal species from two reef habitat types. During this period, thermal anomalies occurred on Palmyra in conjunction with El Niño-Southern Oscillation events in both 2009 and 2015, of which the latter heatwave was longer-lasting and more thermally-severe. We found that coral responses varied by habitat, within and among species, and/or according to the degree of accumulated thermal stress. Nearly all species, particularly *Stylophora pistillata* and *Pocillopora damicornis*, responded more negatively to the 2015 heatwave in terms of colony-specific discoloration and reduction in live planar area. While discoloration was more prominent at the shallower reef terrace compared to the fore reef for this subset of colonies, the reef terrace exhibited greater stability of community-wide coral cover. Colony fate was associated with severity of discoloration at the time of warming: one year following the 2009 heatwave, more severely discolored colonies were more likely to grow, yet following the second heatwave in 2015, colonies were more likely to experience shrinkage or mortality. However, colonies that were more severely discolored in 2009 were not necessarily more discolored in 2015, suggesting that colony-specific factors may be more influential in governing responses to thermal stress.

## Introduction

Climate change and marine heatwaves are increasing the frequency and severity of coral bleaching and mortality events [1, 2], transforming coral assemblages at regional [3–5] and local scales [6, 7]. Climate change, principally ocean warming and acidification, threatens not only coral reef ecosystems’ biodiversity but also their capacity to provide valuable ecological services [8]. Despite evidence that some coral communities can adapt to rising seawater temperatures [9–11], under projected climate scenarios the majority of reefs will likely face repeated and/or annual bleaching events by 2050 [12–14], resulting in extensive reductions in community-wide live coral area as well as shifts in community assemblages toward more stress-tolerant species [15, 16] and acclimatized or heat-adapted individuals [17]. Alternatively, coral reefs may experience local extirpation [18] and phase shifts to non-coral-dominated states [19].

**Fig 1:**
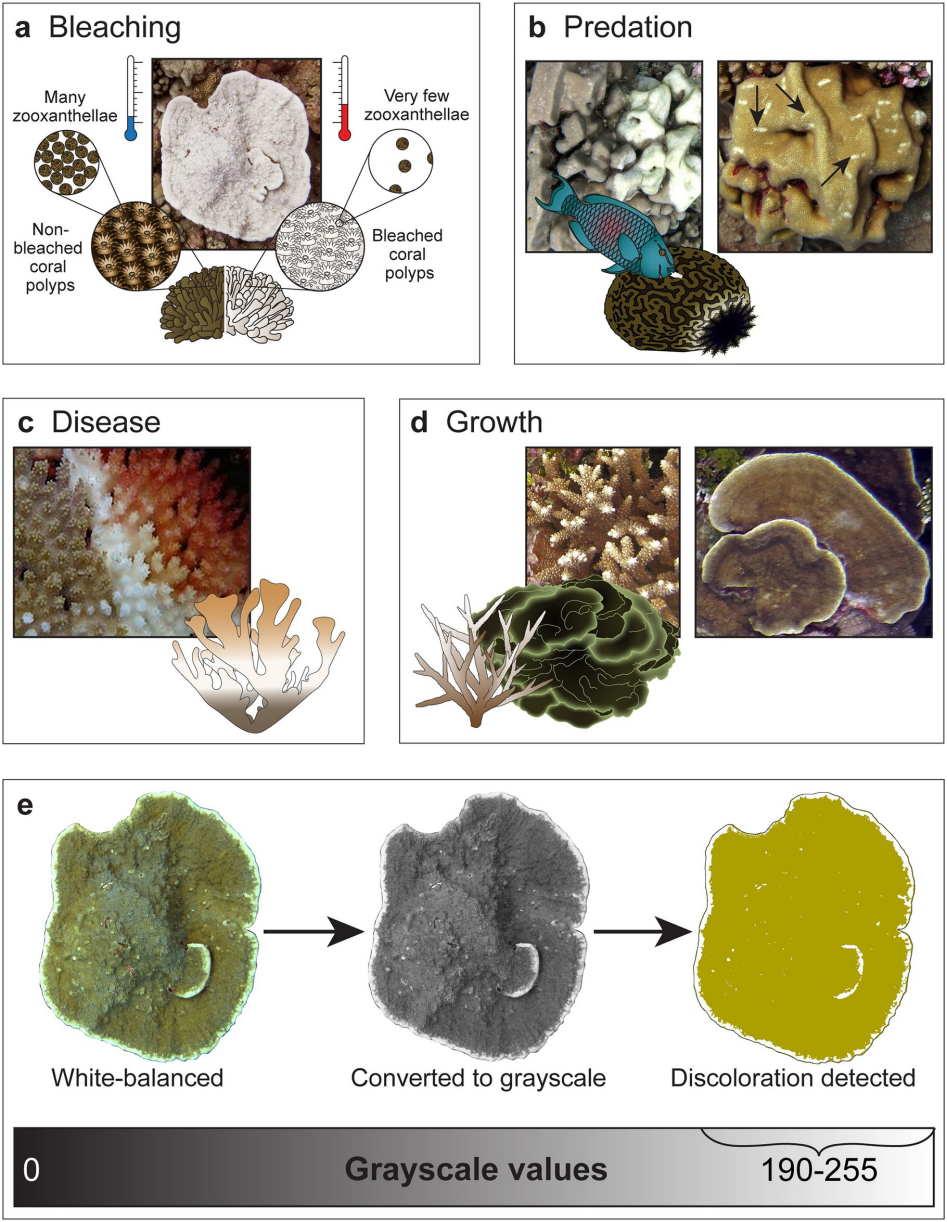
Coral discoloration detection using image analysis. Examples of coral discoloration from Palmyra and their respective causes, resulting from **(a)** elevated seawater temperatures leading to thermal stress (otherwise known as bleaching) or naturally through **(b)** predation, **(c)** disease, or **(d)** growth. An image digitization process for detecting coral discoloration using grayscale (see supporting information) is shown in **(e)**.

Several environmental triggers are known to result in coral bleaching, including reduced salinity, decreased seawater temperature, elevated solar and ultraviolet radiation, or bacterial infection [20]. However, bleaching is typically associated with exposure to anomalous or persistent warm water temperatures, resulting in a breakdown of the relationship between coral hosts and their endosymbiont algae, Symbiodiniaceae (Fig 1a). The susceptibility of corals to thermal stress-induced bleaching differs among coral species and colony morphologies [21–23], which can be linked to the thermal resistance or sensitivity of resident Symbiodiniaceae [24], availability of heterotrophic resources [25], prior history of thermal exposure [26, 27], life history strategies [28], and gene expression or other mechanisms of local adaptation [29, 30].

Corals also experience natural variation in color resulting in paling, patchy coloration, or “discoloration” (i.e., lack of pigmentation not necessarily due to thermal stress). Discoloration can be seen as a result of predation (e.g., by *Drupella* snails or crown-of-thorns sea stars (*Acanthaster*); Fig 1b) or coral disease (such as White Syndrome or White Band Disease; Fig 1c), revealing the skeleton beneath the damaged coral tissue. Additionally, active growth tips or margins of some fast-growing coral taxa (e.g., branching *Acropora* or foliose *Montipora*; Fig 1d) commonly appear white prior to acquiring endosymbionts [31]. Since coloration can be a visual proxy for coral health [32], it would be beneficial to objectively measure a colony’s naturally-occurring change in color over time (Fig 1e) in order to determine how much of the total “bleaching” is actually temperature-induced. Although tools such as the Hawaiian Koʻa Card [33] and the Australian Coral Watch Coral Health Chart [34] are used to assess bleaching in regional score-calibrated cards, they have not been implemented for tracking bleaching within the same colony through time. Since we are quantifying the lack of pigmentation (either naturally-occurring from growth, predation, and disease or caused by thermal stress, without differentiating between them) at every time point—not all of which were thermally anomalous, in the present study we refer to this metric as “discoloration” rather than bleaching.

Many *in situ* observational studies tend to categorize corals as either “fully bleached,” “partially bleached,” or “unbleached” [35–38], however, coral coloration or discoloration encompasses a continuous spectrum and is not a discrete trait. Similarly, distinct color morphs may exist for the same coral species as a result of niche partitioning or symbiont community assemblages [39, 40]. In response to a thermal anomaly, there are various potential outcomes for corals beyond binary survivorship states (i.e., whole-colony survival or mortality), as partial mortality and tissue regeneration are possible [41, 42], particularly for non-acroporids [43]. Current analyses rarely consider the growth trajectories and bleaching proportions of individual coral colonies (but see: [44–46]), whether at the onset of thermal stress (which can range in magnitude and duration) or after peak bleaching has occurred [47]. It has been shown that bleaching and mortality are often more prevalent in areas experiencing higher sea surface temperatures [2, 48]. Thus, when visually evaluating coral condition, it is important to also take into account thermal variability and bleaching chronology [49].

Santavy et al. [50] introduced the term “causal bleaching” to distinguish localized bleaching in individual coral colonies from widespread (i.e., mass) coral bleaching, and similarly, others have suggested differentiating between non- or sublethal bleaching and lethal bleaching [51], yet this approach has not been adopted by the scientific community. Quantifying a given coral colony’s bleaching, paling, or discoloration at multiple points in time and monitoring how this changes in the context of thermal stress would be more informative than single-snapshot surveys, but few such data sets are available. Further, coral responses are known to vary across genera, morphologies, and regions (reviewed in [52]) and each taxon likely has a unique natural baseline of coloration which may fluctuate seasonally and/or with depth, temperature, or other external factors [53, 54]. Colonies of the same taxon may also respond differently [44, 55], and this variability may be attributed to environmental differences such as reef habitat type [56] or local disturbance [57], as well as individual differences such as colony size [58–60], bleaching history [61], or the endosymbiont community structure [62, 63].

In this study, we quantified species-specific, colony-level variability in discoloration (i.e., paling or loss of pigmentation) and colony size (in terms of live planar area) of hard corals on Palmyra Atoll from 2009 to 2019, at least once annually, using image analysis. The goals of this study were to determine if and how coral responses to thermal stress varied by habitat, over time, and/or across two thermal anomalies, and whether some species were more resistant than others with respect to their growth and coloration. We also assessed whether discoloration severity associated with a known thermal anomaly in 2009 and 2015 corresponded to colony fate (i.e., growth, shrinkage, or whole-colony mortality) one year later, and whether colonies that were more discolored during the first heatwave were either more, or less, discolored during the second heatwave. Finally, we explored whether accumulated thermal stress was an accurate predictor of the amount of colony discoloration. We expected to see differential responses within and among species across successive heatwaves, presumably tied to the degree of thermal stress. In an era of accelerated large-scale warming, tracking individual colonies can help to understand why certain corals experience mortality yet others recover, which could better inform management efforts.

## Materials and methods

### Study site

Palmyra (5.89 ºN, 162.08 ºW) is a remote atoll in the Northern Line Islands, located approximately 1,300 km south of Hawai‘i in the central Pacific Ocean. Palmyra is federally-protected as a National Wildlife Refuge as part of the Pacific Remote Island Areas National Marine Monument. Aside from a brief period of military occupation during World War II, Palmyra is uninhabited and experiences minimal direct human impact. As such, its coral reefs are considered quasi-pristine [64], making Palmyra an ideal location to study global change in the absence of confounding local stressors such as fishing and pollution [65]. Palmyra’s reefs are characterized by high benthic cover of hard corals, as well as other reef builders including crustose coralline algae [66, 67], and high herbivore biomass and density [68, 69]. These reefs have been monitored yearly over the past decade, during which they experienced thermal anomalies associated with El Niño-Southern Oscillation (ENSO) in both 2009 and 2015–16 [70, 71].

### Data collection

In 2009, four permanent monitoring sites were established at each of Palmyra’s main reef habitats: the deeper, wave-exposed Fore Reef (FR, 10 m depth), and the shallow, wave-sheltered Reef Terrace (RT, 5 m depth). Ten replicate plots (90 x 60 cm) were established along a 50 m transect line at each site, marked with stainless steel eye bolts in opposing corners and secured with marine epoxy. Photos of the individual plots (i.e., “photoquadrats”) were collected by SCUBA divers using a Canon G Series (G9-G16) camera attached to a tripod with a PVC frame, ensuring that images were taken at a consistent angle and fixed distance from the substrate. Sites were visited at least once per year and up to three times per year from 2009 to 2019. We used image analysis tools in Adobe Photoshop (Creative Cloud) to white-balance the images and determine planar areas of live hard coral colonies, as well as colony-specific discoloration at every time point (see S1 File for detailed methods and justification). Coral colonies were manually assigned identification labels to match the same colonies through time. We also calculated the total percent cover of hard corals in each quadrat and site for each species over time. To compare thermal stress on Palmyra across sampling time points, we used a revised percentile-based method of estimating Degree Heating Weeks (DHW, a measure of accumulated thermal stress; [72]) for central equatorial Pacific reefs developed by Mollica et al. [73]. This metric incorporates inter-annual variability in sea surface temperatures rather than maximum monthly means, since in this region the maximum temperature to which corals are normally exposed does not occur during the same month every year [73]. While elevated percentile-based DHW values were also seen on Palmyra in 2018 and 2019, we considered 2009 and 2015 as thermal anomalies for the purposes of this study because widespread bleaching was only observed *in situ* during those years.

### Statistical analyses

We used non-metric multidimensional scaling (NMDS) via *metaMDS* in the package vegan [74] to visualize the initial and final coral community composition by habitat on Palmyra in September 2009 and September 2019. We calculated Bray-Curtis dissimilarity measures for coral species percent cover in each quadrat, applying a square-root transformation to balance the effect of disproportionately-abundant species [75]. To determine if coral communities varied across space and/or time, we conducted a three-way permutational multivariate analysis of variance (PERMANOVA) with 9999 permutations using *adonis* in vegan, in which habitat and time were considered fixed effects while site (nested within habitat) was considered random. We then identified which species contributed most to these differences through SIMPER or “similarity percentages” analysis using *simper* in vegan [76].

Next, for a subset of nine dominant reef-building coral species whose colonies were entirely present within the photoquadrat frame (*Astrea curta*, *Astreopora myriophthalma*, *Goniastrea stelligera*, *Hydnophora microconos*, *Pavona chiriquiensis*, *Pavona duerdeni*, *Pocillopora damicornis*, *Pocillopora meandrina*, and *Stylophora pistillata*; S1 Table), we examined changes in planar area and discoloration of individual coral colonies through time using image analysis (S1 File). Linear mixed-effects models were used for each species separately to test whether live planar areas or percent discoloration varied over time, by habitat, and/or in response to thermal stress. The models were fitted by restricted maximum likelihood using *lmer* in the lme4 package [77] and assumptions of normality were visually assessed by plotting the residuals. We chose a mixed-effects model instead of a standard repeated measures analysis of variance (ANOVA) because of our unbalanced design, since we did not have observations of all colonies at every time point. Further, the mixed-effects model allowed us to treat time as a continuous variable, given that our time points were not always evenly spaced. Time (months since initial observation in 2009) and habitat were treated as fixed effects (where possible; not all species had representatives from both habitats) and thermal stress (in terms of percentile-based DHW at the time of observation) was treated as a covariate. Colony was considered a random effect to account for repeated measures through time. To identify significant fixed effects, we ran a Type-I ANOVA (or Type-III for interactions between months and habitat) with Satterthwaite’s approximation method on each model. We also investigated whether there was a relationship between accumulated thermal stress and percent discoloration using Pearson’s correlation. To quantify short-term responses, we calculated the percent change in live planar area for individual colonies, averaged by species, one year after each of the thermal anomalies in 2009 and 2015 (i.e., 2010 and 2016, respectively).

Additionally, to explore whether a colony’s level of discoloration during warming corresponded to their fate—colony growth (an increase in live planar area), shrinkage (partial mortality or a reduction in live planar area), or whole-colony mortality, we constructed transition matrices in a format adapted from Williams et al. [78] showing the likelihood of different fates one year following each thermal anomaly. Colonies were grouped into categories based on discoloration severity: 0%, <25%, 25–50%, 51–75%, and >75% (none, low, moderate, high, or severe discoloration) and the number of colonies within a category experiencing each fate was tallied to calculate frequencies and represented through histograms. Due to limited sample sizes, it was not possible to consider colony fate by species, so for the purpose of these transition matrices we combined colonies of the nine focal species. Finally, to determine whether colony fate was dependent on discoloration severity, we used Fisher’s exact test on count data from both heatwaves to compare whether colonies of each discoloration category grew, shrank, or died differently than would be expected by chance [79]. All analyses were conducted in R software version 3.6.3 [80].

## Results

### Coral communities by habitat over time

**Fig 2:**
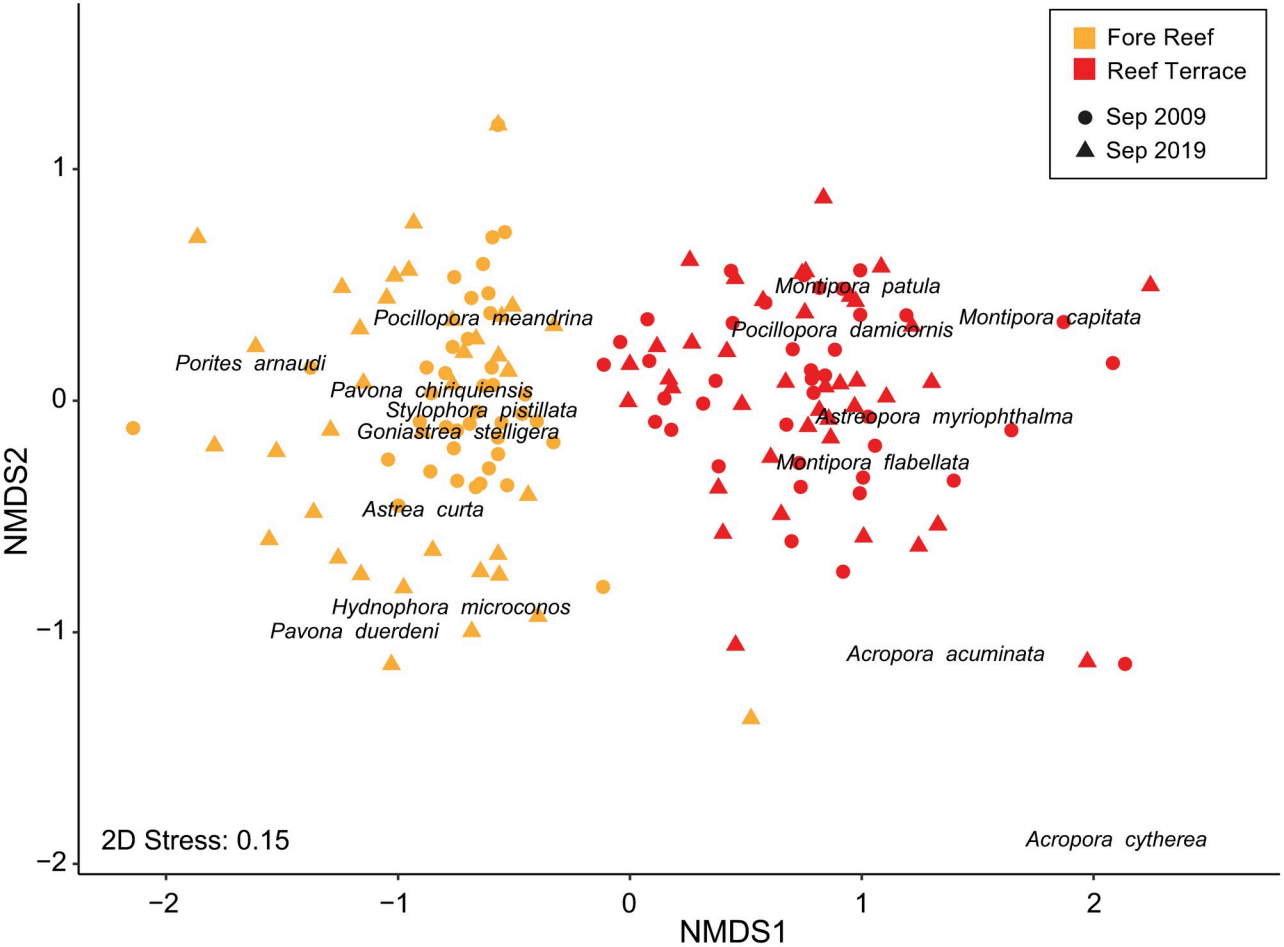
Coral community composition by habitat and year. Non-metric multidimensional scaling (NMDS) based on Bray-Curtis dissimilarity measures of coral community composition by species (in terms of square-root-transformed percent cover data) within each quadrat. Points are color-coded by habitat, with shapes representing different time points in either 2009 or 2019.

Species richness of reef-building hard corals was greater on the fore reef (FR), with at least 29 species seen in photoquadrats taken at this habitat compared to 22 on the reef terrace (RT). Coral community composition at the reef terrace was relatively consistent over time while the fore reef was more dynamic (S3 Fig). Overall, coral cover was higher on the reef terrace at 49.0 ± 0.6% (mean ± SE) and remained stable through time. Coral cover on the fore reef experienced a gradual decline from 35.5 ± 3.0% in 2009 to 17.5 ± 2.3% in 2019. Coral communities were more similar within habitats than among habitats based on Bray-Curtis dissimilarity measures calculated from changes in percent cover at the initial and final time points (Fig 2). From 2009 to 2019, coral community assemblages varied significantly by habitat and site (PERMANOVA, p <0.001; S2 Table), but not over time, and there were no significant interactions. Notably, the coral community at the fore reef was more variable in 2019 than in 2009 (Fig 2). A SIMPER analysis revealed that taxa contributing most to habitat dissimilarity included *Montipora* and *Acropora* spp., predominantly found at the reef terrace, whereas *Pocillopora meandrina*, *Goniastrea stelligera*, and *Porites arnaudi* were more abundant at the fore reef (S3 Table).

### Live area and discoloration in relation to thermal stress

**Fig 3:**
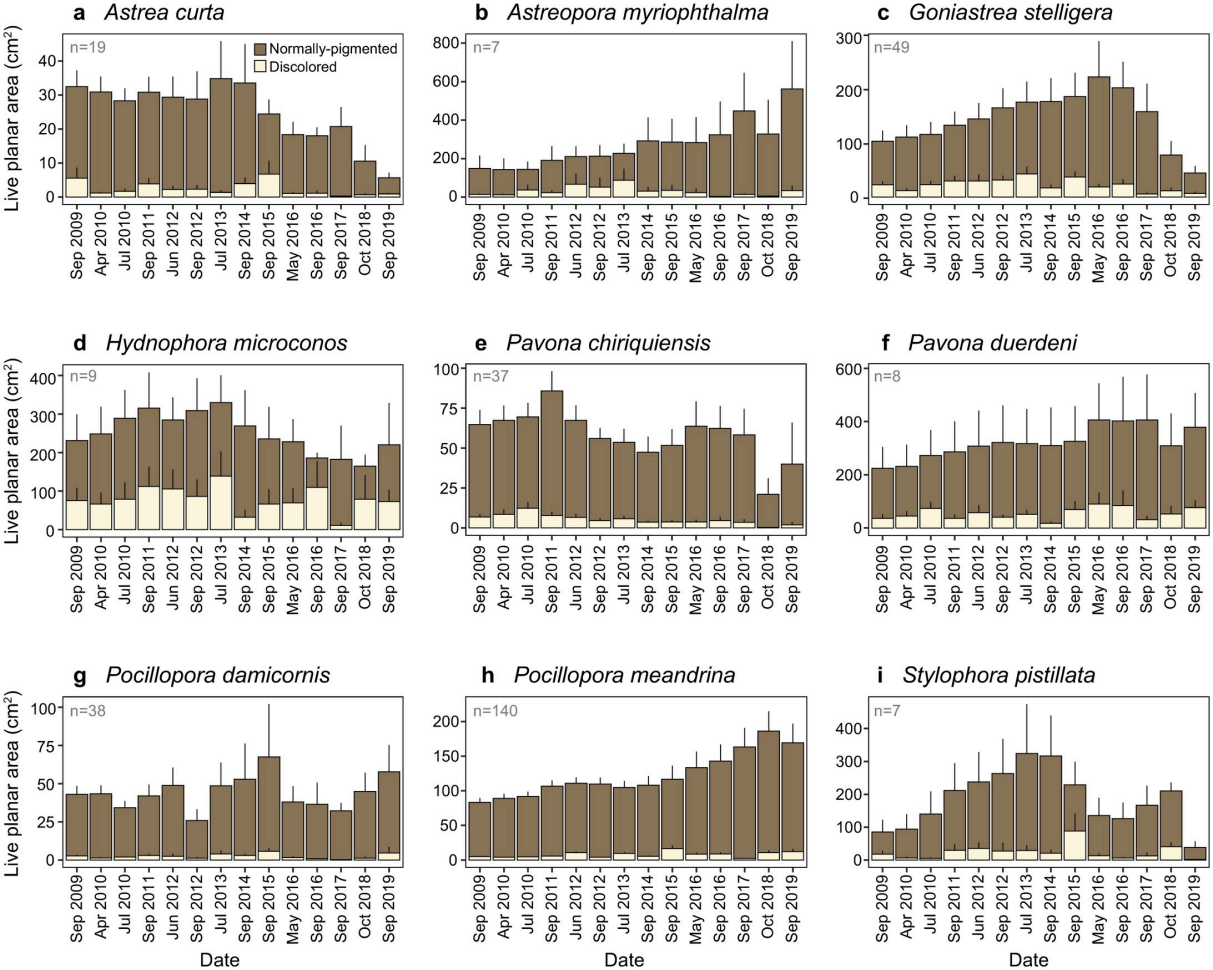
Live planar areas of coral colonies by species over time. Discolored and normally-pigmented live planar areas (mean ± SE) for individual coral colonies over time from both habitats, by species. Colony sample sizes are shown on the top left.

Live planar area of coral colonies varied with thermal stress, by habitat, and/or over time depending on taxon (Fig 3; S4 Table). Percentile-based DHW significantly affected planar area in *A*. *curta*, *H*. *microconos*, *P*. *chiriquiensis*, and *P*. *damicornis*. Colonies of *P*. *damicornis* (n = 38, exclusively from RT) and *A*. *curta* (n = 19, all but one from FR) were the smallest on average at 15.9 ± 1.2 cm^2^ and 16.8 ± 1.3 cm^2^, respectively, while colonies of *P*. *duerdeni* (n = 8 from FR) and *H*. *microconos* (n = 9 from FR) were largest at 236.3 ± 27.4 cm^2^ and 218.8 ± 24.9 cm^2^, respectively. Of species represented at both habitats, colonies of *G*. *stelligera* (n = 34 at FR, n = 15 at RT) and *P*. *meandrina* (n = 116 at FR, n = 24 at RT) were larger at the fore reef (125.7 ± 9.5 cm^2^ and 59.8 ± 2.2 cm^2^, respectively) than the reef terrace (14.5 ± 1.9 cm^2^ and 29.2 ± 1.9 cm^2^, respectively). Live planar area changed significantly through time (p <0.05) for all taxa other than *P*. *duerdeni* and *S*. *pistillata* (S4 Table).

Colonies of *H*. *microconos* and *P*. *duerdeni* experienced the highest discoloration over time at 22.0 ± 2.1% and 21.3 ± 1.6% on average, respectively, while colonies of *P*. *chiriquiensis* and *P*. *damicornis* had the lowest discoloration over time at 9.5 ± 0.7% and 7.3 ± 0.8% on average (S4 Fig). For species found at both habitats, colonies of *G*. *stelligera* and *P*. *meandrina* were more discolored at the reef terrace (31.1 ± 2.3% and 16.2 ± 1.2%, respectively) compared to the fore reef (14.0 ± 0.8% and 6.9 ± 0.4%). Discoloration changed significantly through time for *A*. *myriophthalma* (p = 0.009) and *P*. *chiriquiensis* (p <0.001; S5 Table). Colonies of *S*. *pistillata* (n = 7, only from FR) peaked in discoloration at 40.1 ± 8.7% during the second heatwave in September 2015, the highest among all focal species (S4i Fig). There was a significant effect of percentile-based DHW on percent discoloration in all taxa aside from *A*. *myriophthalma* and *P*. *duerdeni* (S5 Table).

**Fig 4:**
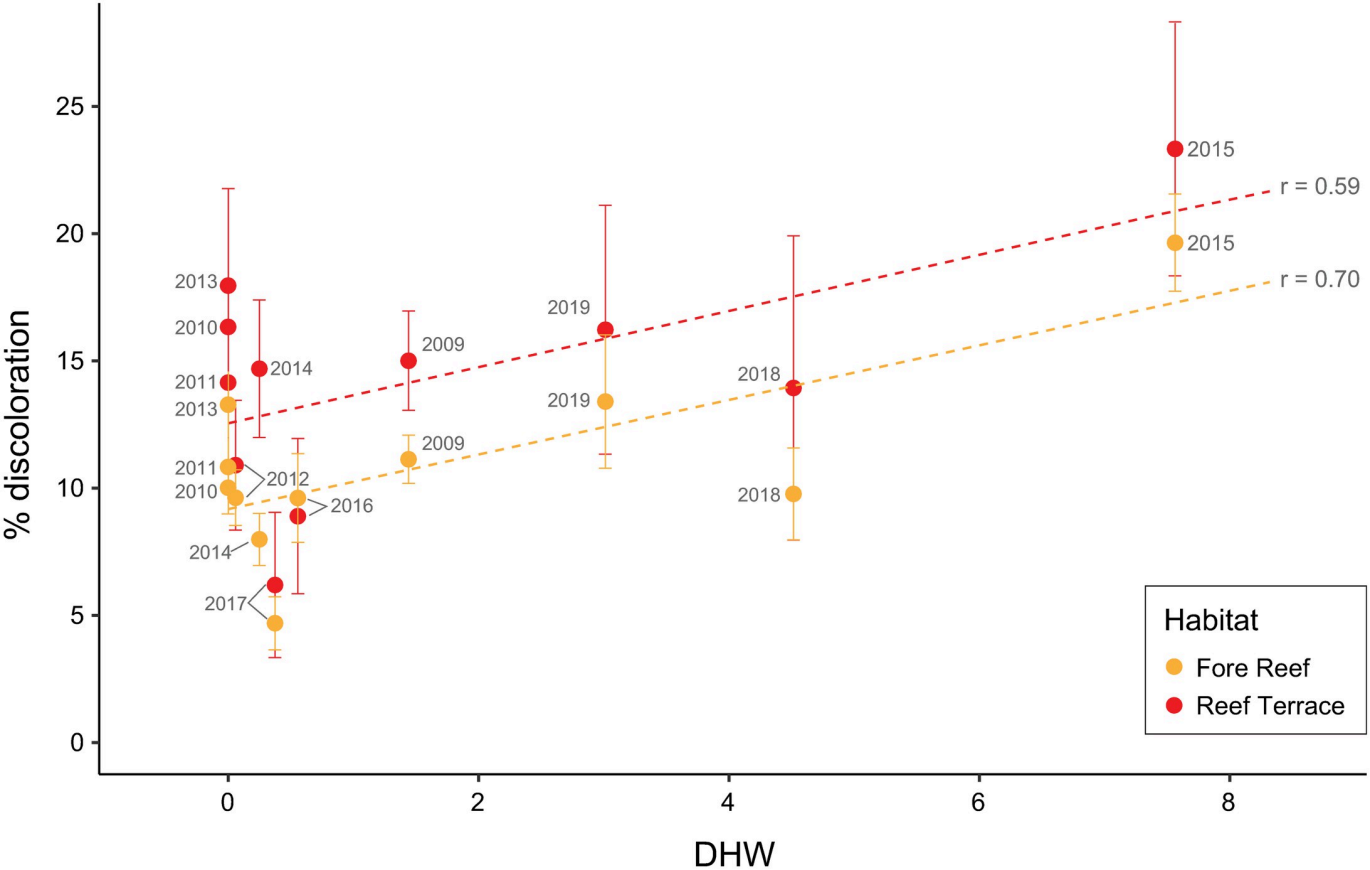
Coral discoloration by habitat and year. Percent discoloration (mean ± SE) by habitat (Fore Reef in orange, Reef Terrace in red) corresponding to the percentile-based Degree Heating Weeks (DHW) at each observation time point, labeled by year. Pearson’s correlation coefficient is shown on the right of the trendlines.

At both habitats, the most discoloration occurred when accumulated thermal stress was at its highest (19.6 ± 1.9% and 23.3 ± 5.0% discoloration at FR and RT, respectively, corresponding to 7.56 DHW in 2015; Fig 4). This correlation was significant for the fore reef (Pearson’s r = 0.70, p = 0.016) but not the reef terrace (Pearson’s r = 0.59, p = 0.055), suggesting that for this data set, percentile-based DHW can be used to reliably predict discoloration only at the fore reef. Discoloration was consistently higher at the reef terrace than the fore reef for this subset of 314 colonies (Fig 4) for species where colony-level analysis was performed.

### Coral colony fate following thermal anomalies

**Fig 5:**
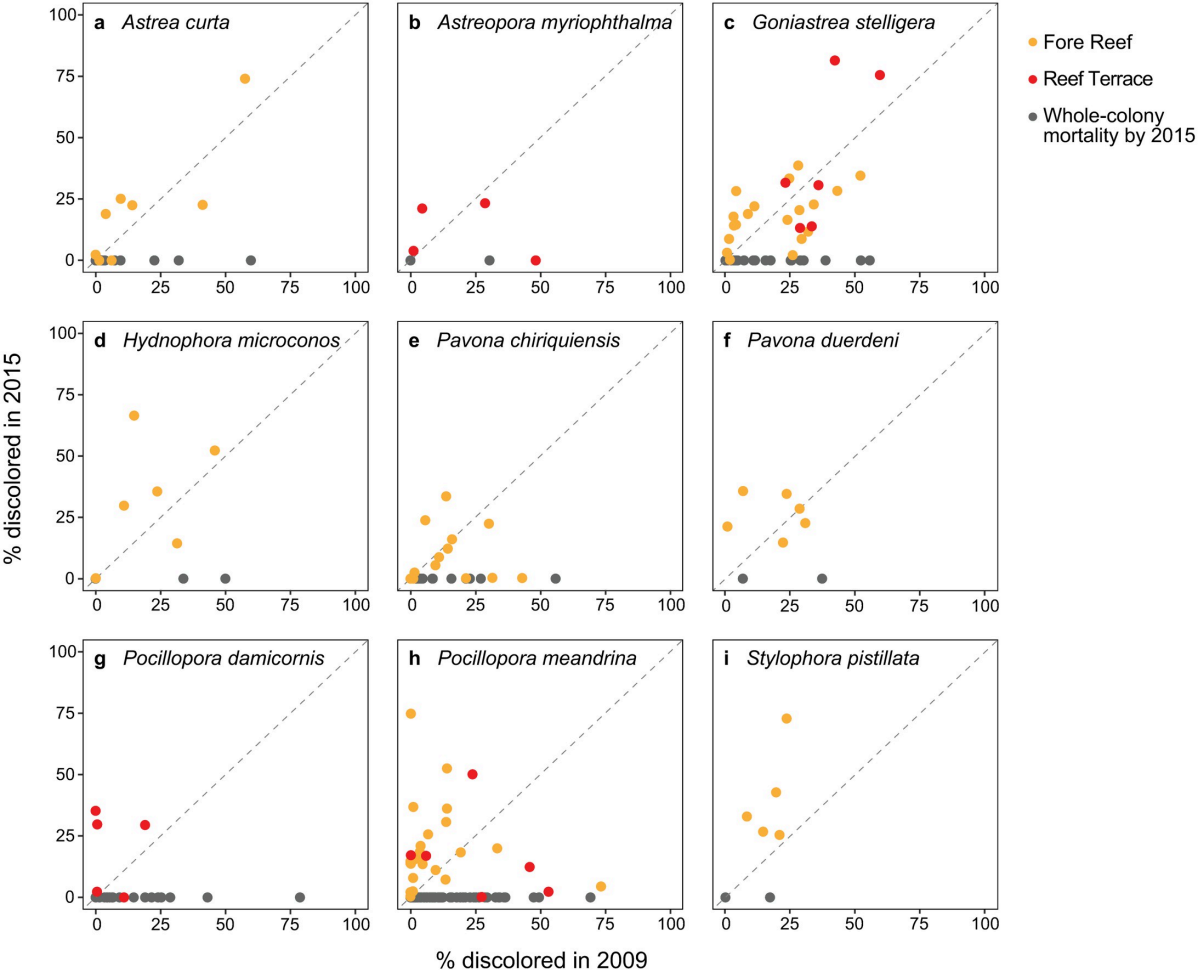
Discoloration comparison for the same coral colonies across two thermal anomalies. Scatterplots comparing percent discoloration in 2009 and 2015 for individual coral colonies by species. Each point represents a coral colony, color-coded by habitat with orange for Fore Reef and red for Reef Terrace. The diagonal dashed line indicates the 1:1 slope in which colonies were discolored by the same amount in both years. If a colony was more discolored in 2015 than 2009, it would fall on the left of the diagonal line and if a colony was less discolored in 2015 than 2009 it would fall to the right of the line. Gray points are colonies that succumbed to whole-colony mortality by 2015.

Percent change in colony planar area also varied by species and thermal anomaly. Palmyra experienced two large-scale thermal anomalies during this study: the first heatwave in 2009 was relatively mild with a percentile-based DHW value of 1.44, while the second heatwave in 2015 was longer-lasting and more thermally-severe at 7.56 DHW [70]. Species whose colonies shrank one year following the first heatwave in 2009 (*P*. *damicornis* and *S*. *pistillata*) lost equal to or more planar area following the second heatwave in 2015 (Table 1). Species whose colonies gained planar area in 2010 (*A*. *curta*, *A*. *myriophthalma*, *G*. *stelligera*, and *H*. *microconos*) shrank in 2016 (Table 1). Colonies of *S*. *pistillata* suffered the highest partial mortality in 2016, while *P*. *duerdeni* colonies exhibited low partial mortality following both heatwaves. When averaging across species, live planar area increased by about 23.4 ± 5.7% after 2009 and decreased by 7.4 ± 5.7% after 2015 (Table 1). Although colonies of *P*. *meandrina* grew following both heatwaves, there were many cases of whole-colony mortality, with only 21.6% of colonies remaining by 2015. Overall, colonies that experienced greater discoloration in 2009 were not necessarily those that experienced whole-colony mortality or greater discoloration in 2015. In fact, most colonies experiencing whole-colony mortality by 2015 were only between 0 to 25% discolored in 2009. For *A*. *curta*, *H*. *microconos*, *P*. *damicornis*, *P*. *meandrina*, and *S*. *pistillata*, discoloration was generally greater in 2015 compared to 2009 (Fig 5). Most colonies of *P*. *chiriquiensis* that survived through 2015 were discolored to a similar extent in 2009 and 2015, while colonies of *G*. *stelligera* had mixed responses.

**Table 1:** Changes in coral colony live planar areas following thermal anomalies.

	1^st^ thermal anomaly		2^nd^ thermal anomaly	
Species	Colony sample size	% change in colony live area one year later (mean ± SE)	Colony sample size	% change in colony live area one year later (mean ± SE)
*Astrea curta*	18	13.5 ± 11.9	8	-8.6 ± 23.7
*Astreopora myriophthalma*	7	18.1 ± 17.6	4	-9.5 ± 16.3
*Goniastrea stelligera*	46	37.3 ± 23.9	25	-28.6 ± 10.1
*Hydnophora microconos*	6	7.6 ± 7.1	6	-13.2 ± 32.2
*Pavona chiriquiensis*	36	11.4 ± 11.5	16	2.3 ± 16.7
*Pavona duerdeni*	8	2.8 ± 20.1	6	-1.6 ± 22.8
*Pocillopora damicornis*	38	-13.2 ± 16.2	5	-17.6 ± 22.8
*Pocillopora meandrina*	139	36.8 ± 7.5	30	11.9 ± 10.9
*Stylophora pistillata*	7	-6.6 ± 25.1	5	-34.8 ± 15.6
Average (all species)	305	23.4 ± 5.7	105	-7.4 ± 5.7

Percent change (mean ± SE) in live planar area for individual colonies by species, one year following thermal anomalies in 2009 and 2015. Positive change (i.e., growth) is shaded in green, negligible change (defined as <5%) in gray, and negative change (i.e., partial mortality) in red. Sample sizes are inconsistent across heatwaves because of some cases of whole-colony mortality by 2015.

As for colony fates, of the 37 colonies with no discoloration in 2009, the majority (73.0%) grew by 2010 (Table 2a, S5a Fig). In contrast, around half (57.1%) of the 7 colonies lacking any discoloration in 2015 grew by 2016 (Table 2b, S5b Fig). In 2009, none of the colonies that were highly (i.e., 51–75%) or severely (>75%) discolored died by 2010, whereas in 2015, severely-discolored colonies either shrank or died by 2016 (although the sample size was low; n = 2). In 2010, shrinkage was most common when colonies had low (<25%) to moderate (25–50%) discoloration the previous year (Table 2a) whereas in 2016, shrinkage was common for colonies of any amount of discoloration (Table 2b). Colonies more severely-discolored in 2009 were less likely to die and more likely to grow by 2010, yet colonies more severely-discolored in 2015 were less likely to grow by 2016. Discoloration severity significantly predicted colony fate after one year (p = 0.03; two-sided Fisher’s exact test). The 2015 heatwave led to more variable responses within and between discoloration categories, although sample sizes were limited for highly or severely-discolored colonies.

**Table 2:** Coral colony fate based on discoloration severity.

	**a**	**Colony fate in 2010**		
		Growth	Shrinkage	Mortality
**Discoloration in 2009**	**0%** (n = 37)	73.0%	16.2%	10.8%
	**<25%** (n = 209)	63.2%	32.1%	4.8%
	**25–50%** (n = 48)	56.3%	35.4%	8.3%
	**51–75%** (n = 10)	90%	10%	0%
	**>75%** (n = 1)	100%	0%	0%
	**b**	**Colony fate in 2016**		
		Growth	Shrinkage	Mortality
**Discoloration in 2015**	**0%** (n = 7)	57.1%	14.3%	28.6%
	**<25%** (n = 60)	50%	43.3%	6.7%
	**25–50%** (n = 24)	41.7%	41.7%	16.7%
	**51–75%** (n = 6)	33.3%	50%	16.7%
	**>75%** (n = 2)	0%	50%	50%

Transition matrices showing the likelihood of different fates for individual coral colonies one year after the **(a)** 2009 and **(b)** 2015 thermal anomalies, based on their discoloration at the time of warming. Colony sample sizes within each discoloration category are indicated in the first column and boxes are shaded according to greater likelihood of outcome. For example, of the 37 colonies that were not at all discolored in 2009, 73.0% of colonies grew the following year while 16.2% of colonies shrank and 10.8% were completely dead by 2010.

## Discussion

Tracking individual coral colonies of different taxa through time may provide insight into species or colony-level traits that promote resistance or recovery from anthropogenic warming. Here we used a photographic approach (Fig 1e; Supp. materials) to quantify colony-level discoloration and changes in live planar area over time for nine reef-building coral species across two reef habitats on Palmyra. We found that the first heatwave in 2009 caused partial mortality only in *Stylophora pistillata* and *Pocillopora meandrina*, while six out of nine species responded negatively to the second heatwave in 2015 (Table 1). Overall, for the 314 individual coral colonies, discoloration was higher at the reef terrace than the fore reef (Fig 4) throughout the time series; however, from 2009 to 2019, community-wide coral cover declined on the fore reef whereas it was maintained at the reef terrace (S3 Fig), showing a higher capacity for resilience at this habitat.

Among the most discolored species in 2015 was the branching coral *Stylophora pistillata* (S4i Fig), which was previously found to have the highest bleaching prevalence and severity on Palmyra in 2009 [71]. *Stylophora pistillata* is relatively fast-growing and short-lived (i.e., “weedy”), as are many *Pocillopora* spp. [28], which had low bleaching prevalence in 2009 [71] and minimal discoloration through time (Fig 3g and 3h). Although we saw many cases of whole-colony mortality in branching *Pocillopora* (Fig 5g and 5h), this genus is known for its high recruitment density and population turnover [81] and new individuals were established across the 11-year time span. *Goniastrea stelligera* and *Astrea curta*, both massive/sub-massive species which are generally thought to be more resistant to bleaching [21, 23], showed higher bleaching severity on Palmyra in 2009 [71] with low to moderate discoloration through time, and colonies ultimately lost much of their live area (Fig 3a–3c). In contrast, colonies of *Hydnophora microconos* were naturally more discolored than other species (but see [82] regarding their high within-colony Symbiodiniaceae diversity and flexibility), yet their live area was similar on average between final and initial time points (Fig 3d). Massive/encrusting *Pavona* spp., previously noted for their stress tolerance and fast recovery rates [83, 84], had low discoloration and were more stable in terms of their planar area one year post-heatwave (Table 1). Meanwhile, colonies of encrusting *Astreopora myriophthalma*, which also showed the lowest bleaching prevalence in 2009 [71] grew by 2019 (Fig 3b), highlighting variability in responses among taxa and morphologies.

Coral community structure and bleaching responses on Palmyra are also known to vary by habitat [67] (yet this does not seem to drive symbiont distribution patterns; [85]), with more subsequent mortality on the fore reef and limited mortality on the reef terrace [70]. As such, cover of hard corals has been gradually declining on Palmyra’s fore reef since 2015 [66] yet has remained consistent at the reef terrace as of 2019. Past studies have similarly found that abiotic and biotic factors influence local bleaching prevalence [71, 86, 87]. Solar heating and irradiance is higher at the shallower, wave-sheltered reef terrace [67], which could explain why seasonal paling or discoloration was more prominent at this habitat in the present study. Deeper, more turbid waters are thought to provide a refuge for corals due to the attenuation of heat or light [53, 88], but depth does not always confer resistance to bleaching [89, 90] and there is evidence that some taxa may benefit from a depth refuge more than others [54]. In the present study, *P*. *meandrina* and *G*. *stelligera* colonies experienced less discoloration on the deeper, wave-exposed fore reef. On Palmyra, the potential for a thermal refuge driven by upwelling is seen below 15 m depth [91] but given that all of our sites were relatively shallow (5 m or 10 m depth), effects of cooling were likely limited. Other environmental variables that differ by habitat on Palmyra include temperature [70], sedimentation and turbidity [71], hydrodynamic connectivity [92], pH and dissolved oxygen fluxes [93, 94], and heterotrophic resource availability [95]. Corals on the reef terrace experience more diurnal variability, which may have prepared them to withstand the effects of thermal stress. Reef terrace corals also receive inputs of zooplankton and particulate organic matter from the nearby lagoon, which may serve as a source of heterotrophic nutrition when autotrophy by endosymbionts is compromised. In particular, *P*. *meandrina* colonies on Palmyra have shown a high degree of trophic plasticity [25] and this may further contribute to their resistance during bleaching [70].

The intensity of accumulated thermal stress at the time of each heatwave corresponded to the amount of discoloration or bleaching, which aligns with findings from previous studies [96, 97]. Contextualizing discoloration severity against percentile-based DHWs allowed us to examine natural variations in discoloration and how this may deviate from baseline levels. In our study, thermal-stress associated bleaching and natural paling were combined into one metric, but perhaps there would have been a stronger relationship between thermal stress and colony fate if we had distinguished between them. Nevertheless, a colony’s extent of discoloration at the time of warming was associated with its fate one year later; more severe discoloration corresponded to a higher likelihood of growth following the first heatwave and a lower likelihood of growth following the second heatwave (Table 2). Previous studies have similarly found a positive association between bleaching prevalence and subsequent colony mortality [44, 78], but our results further indicate that these responses may differ across successive heatwaves and/or according to the degree of accumulated thermal stress.

Following the first, less thermally-severe heatwave in 2009, a positive change in planar area (i.e., growth) was the most common outcome in 2010 for all colonies regardless of their discoloration severity yet following the longer-lasting, more thermally-severe heatwave in 2015, colony fates were more variable. Colonies with high to severe discoloration in 2015 were less likely to grow in 2016, with many colonies experiencing shrinkage or whole-colony mortality. However, colonies that were more discolored in 2015 were not necessarily those that were more discolored in 2009 (Fig 5), which we might expect if we assume that a colony is less resistant when faced with repeated bleaching [26, 49, 98]. Interestingly, most species that gained planar area in response to the first anomaly shrank after the second anomaly, while species that declined in planar area in 2010 lost a similar or greater amount of live tissue in 2016 (Table 1). On the contrary, if colonies were less discolored in 2015 compared to 2009, this could indicate heightened thermal tolerance or acclimatization [99]. Perhaps under stressful conditions, a colony’s fate depends more so on other taxon and colony-specific characteristics (e.g., larger colony size, more thermally-tolerant symbionts, environmental “legacy” or physiological history, biomass reserve quality and quantity). With large-scale disturbances occurring more frequently due to climate change, these inter- and intraspecific differences may become more critical in modulating bleaching susceptibility.

One limitation of our study is that we could not measure the responses of all common coral species on Palmyra at the colony level, nor look more closely at habitat or site-specific patterns due to the lack of representatives. When using imagery to measure the growth and discoloration of the same individual colonies through time, ideally the entire colony is in view; however, in our small-scale images (0.54 m^2^ quadrats), colonies were often cut off by the photoquadrat frame and excluded from colony-level analyses, which constrained our sample sizes. Large-area imagery (e.g., 100 m^2^ photomosaics as in [100, 101]) might be better suited for comprehensive analyses, although methods for quantifying bleaching proportions in individual colonies using this technology have not yet been developed. Further, assessing coral health visually through imagery does not take into account physiological traits such as symbiont type, density, and pigment concentrations nor reliance on heterotrophy. For a better-informed view of post-bleaching responses, physiological data must be incorporated in addition to (dis)coloration.

Overall, this study demonstrates variability in coral responses by habitat, through time, and among and within taxa in the context of thermal anomalies, emphasizing a need for more precise longer-term monitoring at the species and individual colony level. Tracking parameters such as growth, bleaching or discoloration, recovery, and partial or whole-colony mortality in the same colonies at multiple points in time will improve our knowledge of the effects of cumulative thermal stress on coral communities and their constituent taxa. Documenting the natural history of quasi-pristine, intact ecosystems such as Palmyra Atoll allows us to establish baseline information on coral trajectories under global stressors, which is becoming increasingly relevant for the conservation and restoration of more-degraded reefs at higher risk of collapse.

## Supporting information

S1 File Detailed methods for detecting coral discoloration using image analysis. (DOCX)

S1 Fig Justification for choosing a grayscale range in detecting coral discoloration. Boxplots comparing the discoloration detected semi-automatically using various grayscale ranges to “by-eye” (i.e., human-designated) discoloration.
(TIF)

S2 Fig Justification for choosing a photo-editing method in detecting coral discoloration. Boxplots comparing the discoloration detected semi-automatically using various photo-editing methods to “by-eye” (i.e., human-designated) discoloration.
(TIF)

S3 Fig Benthic coral cover by species and habitat through time. Mean percent benthic cover of hard corals (averaged across sites) at the **(a)** Fore Reef and **(b)** Reef Terrace habitats on Palmyra, by species, over time.
(TIF)

S4 Fig Percent discoloration of coral colonies by species over time. Percent discoloration (mean ± SE) for individual coral colonies over time by species and habitat, with Fore Reef (FR) in orange and Reef Terrace (RT) in red. Colony sample sizes are shown on the top left. Dashed vertical lines indicate thermal anomalies in 2009 and 2015.
(TIF)

S5 Fig Coral colony fate based on discoloration severity. Histograms showing the number of colonies from each discoloration category experiencing growth (in green), shrinkage (yellow), or whole-colony mortality (red) one year following the **(a)** 2009 and **(b)** 2015 thermal anomalies.
(TIF)

S1 Table Sample sizes for colony-specific analyses by species at each habitat. Overall, 314 individual colonies from nine different species were tracked in total. Only colonies that were fully within the photoquadrat frame were included in colony-level analyses.
(DOCX)

S2 Table PERMANOVA results for coral communities by habitat and site over time. Statistical output from a three-way permutational analysis of variance (PERMANOVA, 9999 permutations) on Bray-Curtis dissimilarities for square root-transformed coral species cover data by habitat, time point, site (nested within habitat), and their interactions. Bold indicates statistical significance (α = 0.05).
(DOCX)

S3 Table SIMPER results for coral communities by habitat. SIMPER (similarity percentage) analysis output identifying the species contributing most to community composition differences between habitats.
(DOCX)

S4 Table ANOVA results for live planar area of coral colonies by species. Statistical output from a Type-I (or Type-III for interactions) analysis of variance (ANOVA) for the effects of Degree Heating Weeks (DHW), month, and/or habitat on live planar area of individual coral colonies, by species. Bold indicates statistical significance (α = 0.05).
(DOCX)

S5 Table ANOVA results for percent discoloration of coral colonies by species. Statistical output from a Type-I (or Type-III for interactions) analysis of variance (ANOVA) for the effects of Degree Heating Weeks (DHW), month, and/or habitat on percent discoloration of individual coral colonies, by species. Bold indicates statistical significance (α = 0.05).
(DOCX)
