# Supplementary material for: Inter- and intraspecific responses of coral colonies to thermal anomalies on Palmyra Atoll, central Pacific
Source: PLoS One. 2024 Nov 25;19(11):e0312409. doi: 10.1371/journal.pone.0312409 (PMC11588205; doi:10.1371/journal.pone.0312409)
Supplement: S1 File — (DOCX) [file pone.0312409.s001.docx]

*Image Digitization Methods*

Images were analyzed entirely in Adobe Photoshop (Creative Cloud). First, the raw images were white-balanced using the whitest point on the photoquadrat PVC frame. The borders of all live coral colonies were traced manually with the pencil tool (2-pixel width, in black) on a duplicate image layer. The paint bucket was then used to fill in each coral colony with the corresponding genus/species swatch color to the finest possible taxonomic resolution. Individual coral colonies of all species were each assigned an identification number and digitally labeled for tracking the same colonies’ changes in planar areas throughout the time series. Next, while having coral areas selected via the magic wand, the color range command in Photoshop was used to detect “highlights,” otherwise known as the discolored surface coral tissue. Essentially, this tool is converting the entire image (or selected regions) to grayscale to isolate only certain pixels based on a predetermined brightness threshold (Fig 1e). Fuzziness was set to 0% and range was set to 190; the Fuzziness setting increases or decreases the number of partially-selected pixels and range (i.e., of brightness) is measured on a scale from 0 (black) to 255 (white). If range is set to 255, it will select pure white (i.e., totally bleached) pixels; if range is set to ~190, it will also select partially-bleached or discolored pixels. We chose 190 because this range of values (190-255) has been proven to be most comparable to a human’s by-eye bleaching designations; see further justification below.

Discoloration was filled in with solid white on another image layer using the paint bucket; however, if for some reason the computer was not accurate in its designations, we manually corrected them. Discrepancies were largely caused by unfavorable lighting conditions at the time the pictures were taken; for example, in the case of overexposure, the computer may have detected more artificial “whiteness,” which the human would erase accordingly. Alternatively, if corals were under a shadow at the time when the picture was taken and thus discoloration was not picked up by the computer, the human would consult their judgment and add in those areas by hand. Thus, although discoloration detection is carried out semi-automatically using Photoshop-based tools, it ultimately relies on human expertise, allowing for efficiency and objectivity as well as accuracy.

Data were extracted directly in Photoshop by first using the magic wand tool to select the color that corresponded to the corals being measured, one species or colony at a time. The ‘contiguous’ setting was turned on (this will select only pixels that are directly touching one another). Tolerance was set to 0 (only selecting pixels of the same exact color value). Next, the image analysis tool was used to find total planar area measurements of each live coral colony as well as discoloration within that colony. The measurement scale was set to custom and the ruler was placed along the distance between the inner corners of either the long or short edge of the photoquadrat frame. The logical length was either 90 or 60 (for the long or short edge, respectively), and the logical units were centimeters. When generating measurements, Photoshop converts the pixel counts to planar areas in cm^2^. This process was repeated for each coral colony, recording both the total (live) and discolored planar areas; the difference between them is the “normally-pigmented” (i.e., non-discolored) coral planar area.

*Discoloration Detection: Justification for Choosing a Grayscale Range*

Although the grayscale range 190-255 for detecting coral discoloration is seemingly arbitrary, the lower limit of 190 has been tested against every other possible value between 165 and 215, in increments of 5, for discolored pixel counts within all coral colonies from a random subset of images (n = 58 images total from several sites and time points) that were analyzed both “by-eye” (i.e., derived from a human expert’s hand-tracings, assuming these are the most trustworthy) and semi-automatically using Photoshop. Results showed that having 190 as the lower limit of the range leads to a nearly negligible difference on average from what would be selected “by-eye” (S1 Fig). Any value below or above 190 would, respectively, over- or underestimate discoloration.

*Discoloration Detection: Justification for Choosing a Photo-Editing Method*

Similarly, we must also recognize that the photo-editing method on a raw image (e.g., unedited, color-corrected, or white-balanced) will affect the brightness of the image, which in turn could yield slightly different discoloration amounts detected by Photoshop. As such, we have compared the “by-eye” (human-designated) discolored pixel counts to those obtained semi-automatically in Photoshop for the same subset of images. Results showed that either color-corrected or white-balanced images are the most similar to “by-eye” on average (S2 Fig). However, considering that we are measuring discoloration for the same colonies over time, we want to avoid the outliers (likely due to overexposure) associated with color-correcting the image. Thus, white-balancing the images was chosen as the appropriate photo-editing method because it most closely matches “by-eye” designations with relatively fewer outliers.
