# Supplementary figures and images for: Inter- and intraspecific responses of coral colonies to thermal anomalies on Palmyra Atoll, central Pacific
Source: PLoS One. 2024 Nov 25;19(11):e0312409. doi: 10.1371/journal.pone.0312409 (PMC11588205; doi:10.1371/journal.pone.0312409)

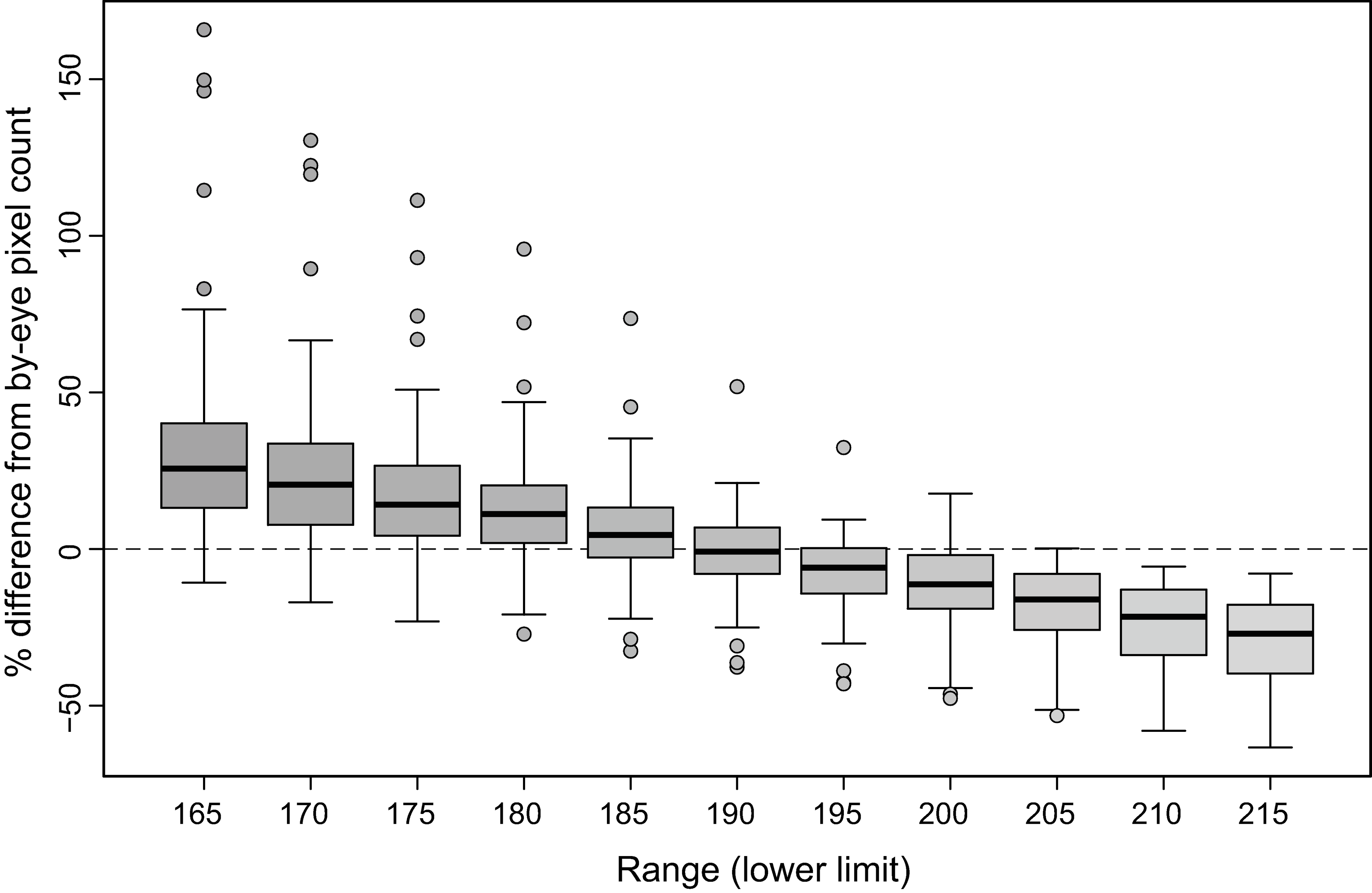

Supplement: S1 Fig — Boxplots comparing the discoloration detected semi-automatically using various grayscale ranges to “by-eye” (i.e., human-designated) discoloration. (TIF) [file pone.0312409.s002.tif]

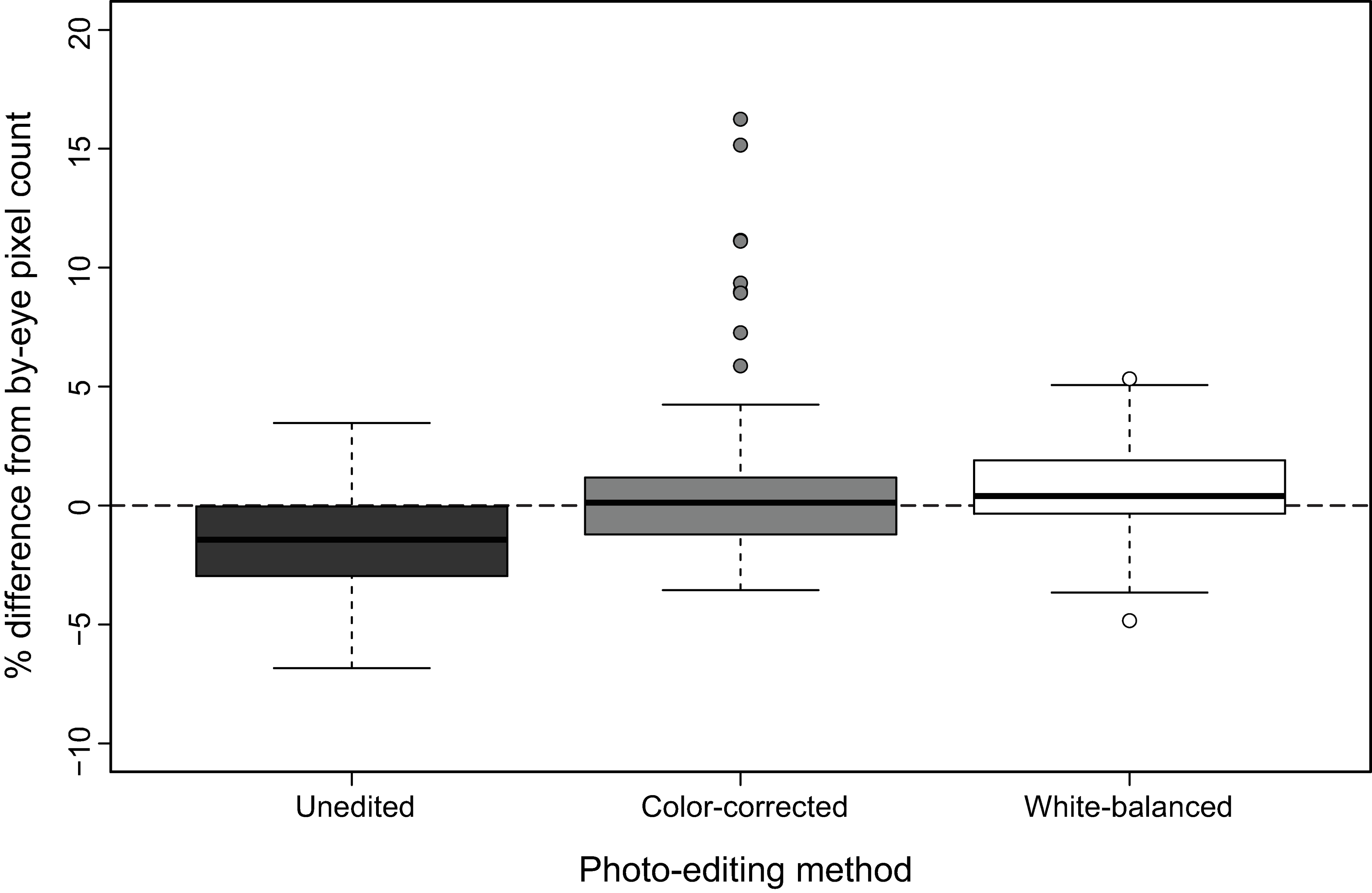

Supplement: S2 Fig — Boxplots comparing the discoloration detected semi-automatically using various photo-editing methods to “by-eye” (i.e., human-designated) discoloration. (TIF) [file pone.0312409.s003.tif]

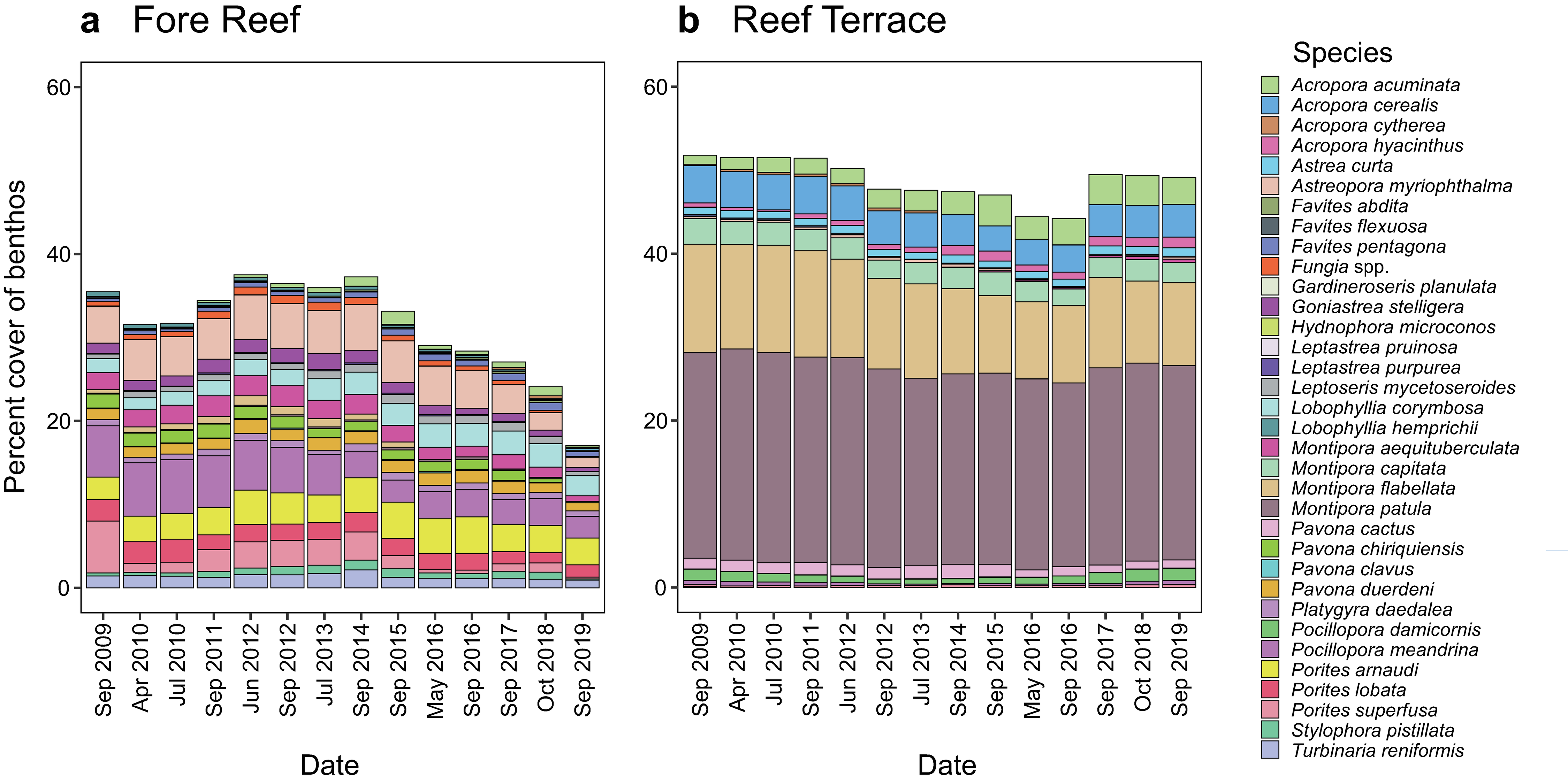

Supplement: S3 Fig — Mean percent benthic cover of hard corals (averaged across sites) at the (a) Fore Reef and (b) Reef Terrace habitats on Palmyra, by species, over time. (TIF) [file pone.0312409.s004.tif]

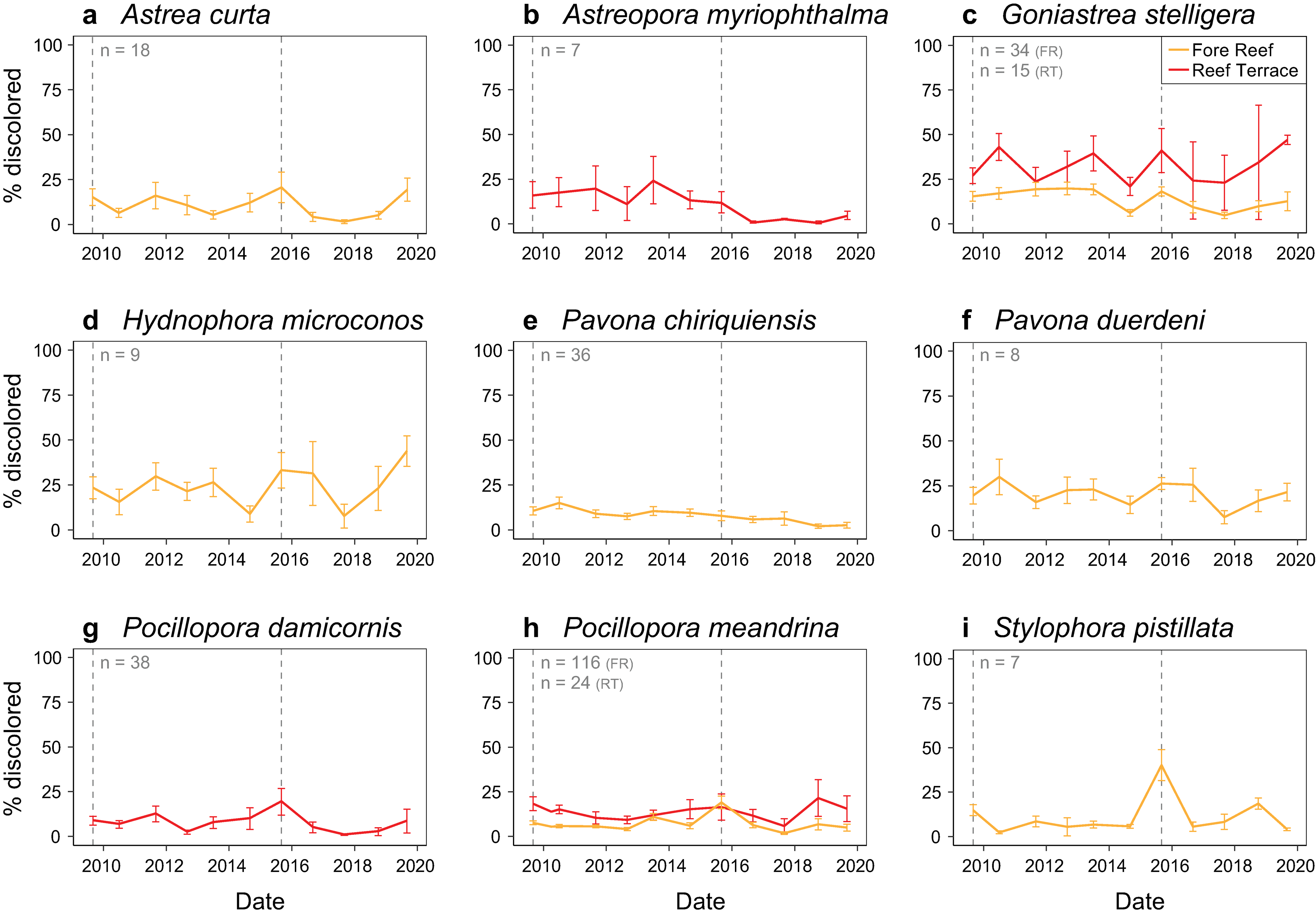

Supplement: S4 Fig — Percent discoloration (mean ± SE) for individual coral colonies over time by species and habitat, with Fore Reef (FR) in orange and Reef Terrace (RT) in red. Colony sample sizes are shown on the top left. Dashed vertical lines indicate thermal anomalies in 2009 and 2015. (TIF) [file pone.0312409.s005.tif]

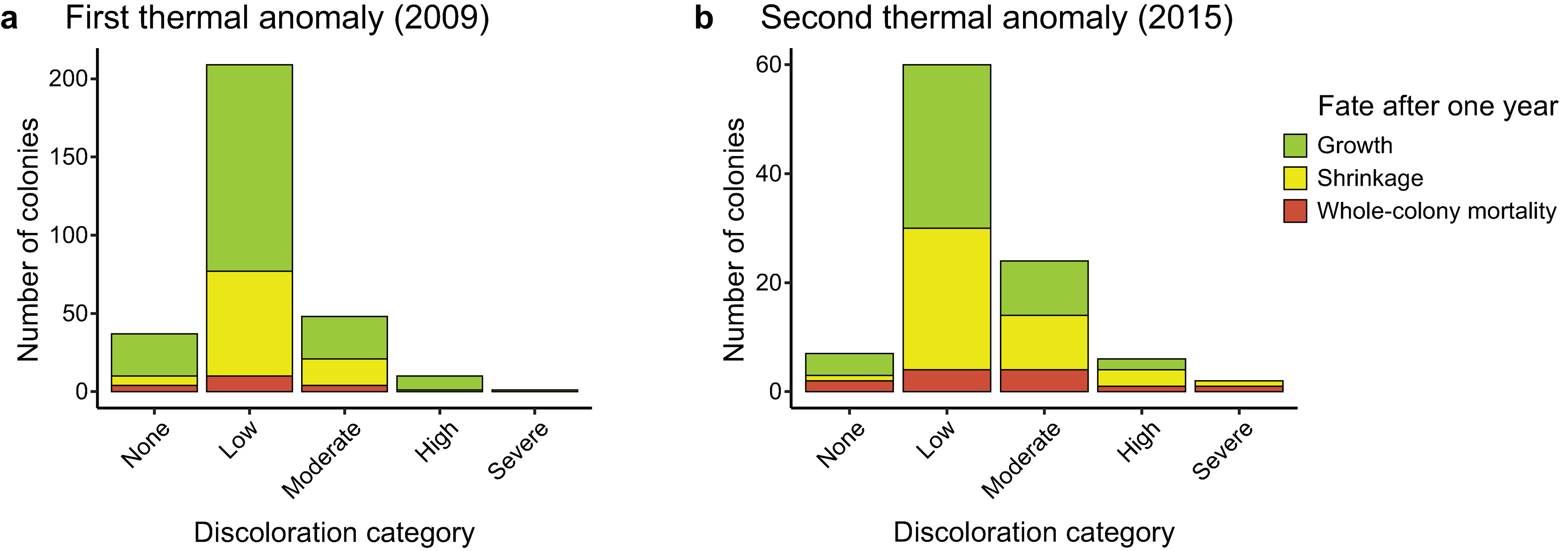

Supplement: S5 Fig — Histograms showing the number of colonies from each discoloration category experiencing growth (in green), shrinkage (yellow), or whole-colony mortality (red) one year following the (a) 2009 and (b) 2015 thermal anomalies. (TIF) [file pone.0312409.s006.tif]
