# Supplementary material for: Inter- and intraspecific responses of coral colonies to thermal anomalies on Palmyra Atoll, central Pacific
Source: PLoS One. 2024 Nov 25;19(11):e0312409. doi: 10.1371/journal.pone.0312409 (PMC11588205; doi:10.1371/journal.pone.0312409)
Supplement: S1 Table — Overall, 314 individual colonies from nine different species were tracked in total. Only colonies that were fully within the photoquadrat frame were included in colony-level analyses. (DOCX) [file pone.0312409.s007.docx]

**S1 Table.** **Sample sizes for colony-specific analyses by species at each habitat.**

| Species | Fore Reef (FR) | Reef Terrace (RT) | Total |
| --- | --- | --- | --- |
| *Astrea curta* | 18 | 1 | 19 |
| *Astreopora myriophthalma* | 0 | 7 | 7 |
| *Goniastrea stelligera* | 34 | 15 | 49 |
| *Hydnophora microconos* | 9 | 0 | 9 |
| *Pavona chiriquiensis* | 36 | 1 | 37 |
| *Pavona duerdeni* | 8 | 0 | 8 |
| *Pocillopora damicornis* | 0 | 38 | 38 |
| *Pocillopora meandrina* | 116 | 24 | 140 |
| *Stylophora pistillata* | 7 | 0 | 7 |
| Grand total | 228 | 86 | 314 |

Overall, 314 individual colonies from nine different species were tracked in total. Only colonies that were fully within the photoquadrat frame were included in colony-level analyses.
