# Supplementary material for: Inter- and intraspecific responses of coral colonies to thermal anomalies on Palmyra Atoll, central Pacific
Source: PLoS One. 2024 Nov 25;19(11):e0312409. doi: 10.1371/journal.pone.0312409 (PMC11588205; doi:10.1371/journal.pone.0312409)
Supplement: S2 Table — Statistical output from a three-way permutational analysis of variance (PERMANOVA, 9999 permutations) on Bray-Curtis dissimilarities for square root-transformed coral species cover data by habitat, time point, site (nested within habitat), and their interactions. Bold indicates statistical significance (α = 0.05). (DOCX) [file pone.0312409.s008.docx]

**S2 Table.** **PERMANOVA results for coral communities by habitat and site over time.**

| Source | Df | SumsSqs | MeanSqs | F.Model | R^2^ | Pr(>F) |
| --- | --- | --- | --- | --- | --- | --- |
| Habitat | 1 | 110.52 | 110.523 | 417.63 | 0.240 | **<0.001** |
| Date | 16 | 5.07 | 0.317 | 1.20 | 0.011 | 0.066 |
| Site(Habitat) | 6 | 45.67 | 7.611 | 28.76 | 0.099 | **<0.001** |
| Habitat * Date | 15 | 4.43 | 0.296 | 1.12 | 0.010 | 0.168 |
| Site(Habitat) * Date | 84 | 7.86 | 0.094 | 0.35 | 0.017 | 1.000 |
| Residuals | 1084 | 286.88 | 0.265 |  | 0.623 |  |
| Total | 1206 | 460.43 |  |  | 1.000 |  |

Statistical output from a three-way permutational analysis of variance (PERMANOVA, 9999 permutations) on Bray-Curtis dissimilarities for square root-transformed coral species cover data by habitat, time point, site (nested within habitat), and their interactions. Bold indicates statistical significance (𝛼 = 0.05).
