# Supplementary material for: Inter- and intraspecific responses of coral colonies to thermal anomalies on Palmyra Atoll, central Pacific
Source: PLoS One. 2024 Nov 25;19(11):e0312409. doi: 10.1371/journal.pone.0312409 (PMC11588205; doi:10.1371/journal.pone.0312409)
Supplement: S3 Table — SIMPER (similarity percentage) analysis output identifying the species contributing most to community composition differences between habitats. (DOCX) [file pone.0312409.s009.docx]

**S3 Table.** **SIMPER results for coral communities by habitat.**

| Species | Avg  abundance at FR | Avg abundance at RT | Avg contribution to overall dissimilarity (± SD) | Cumulative contribution  (%) |
| --- | --- | --- | --- | --- |
| *Montipora patula* | 0.155 | 22.293 | 0.288 (± 0.249) | 29.63 |
| *Montipora flabellata* | 0.491 | 10.667 | 0.147 (± 0.183) | 44.787 |
| *Pocillopora meandrina* | 4.357 | 0.298 | 0.059 (± 0.063) | 50.866 |
| *Goniastrea stelligera* | 4.174 | 0.214 | 0.054 (± 0.066) | 56.42 |
| *Porites arnaudi* | 3.431 | 0 | 0.042 (± 0.083) | 60.782 |
| *Acropora cytherea* | 0 | 4.012 | 0.037 (± 0.156) | 64.572 |
| *Acropora acuminata* | 0.449 | 2.461 | 0.037 (± 0.113) | 68.335 |
| *Montipora capitata* | 0 | 2.378 | 0.036 (± 0.097) | 72.087 |

SIMPER (similarity percentage) analysis output identifying the species contributing most to community composition differences between habitats.
