# Supplementary material for: Inter- and intraspecific responses of coral colonies to thermal anomalies on Palmyra Atoll, central Pacific
Source: PLoS One. 2024 Nov 25;19(11):e0312409. doi: 10.1371/journal.pone.0312409 (PMC11588205; doi:10.1371/journal.pone.0312409)
Supplement: S4 Table — Statistical output from a Type-I (or Type-III for interactions) analysis of variance (ANOVA) for the effects of Degree Heating Weeks (DHW), month, and/or habitat on live planar area of individual coral colonies, by species. Bold indicates statistical significance (α = 0.05). (DOCX) [file pone.0312409.s010.docx]

**S4 Table.** **ANOVA results for live planar area of coral colonies by species.**

| Species | Source | SumSq | Df  (num, den) | F value | Pr(>F) |
| --- | --- | --- | --- | --- | --- |
| *Astrea curta* | DHW | 5452.6 | 1, 259.01 | 23.565 | **<0.001** |
|  | Months | 19413.4 | 1, 260.12 | 83.902 | **<0.001** |
| *Astreopora myriophthalma* | DHW | 23206 | 1, 100 | 3.372 | 0.069 |
|  | Months | 62528 | 1, 100 | 9.086 | **0.003** |
| *Goniastrea stelligera* | DHW | 5477 | 1, 668.05 | 1.217 | 0.270 |
|  | Months | 196932 | 1, 668.07 | 43.773 | **<0.001** |
|  | Habitat | 40954 | 1, 49.33 | 9.103 | **0.004** |
|  | Months * Habitat | 135064 | 1, 668.07 | 30.021 | **<0.001** |
| *Hydnophora microconos* | DHW | 248911 | 1, 118.06 | 7.558 | **0.007** |
|  | Months | 475926 | 1, 118.09 | 14.452 | **<0.001** |
| *Pavona chiriquiensis* | DHW | 93244 | 1, 523.15 | 93.217 | **<0.001** |
|  | Months | 217809 | 1, 523.35 | 217.745 | **<0.001** |
| *Pavona duerdeni* | DHW | 11974.7 | 1, 112 | 1.474 | 0.227 |
|  | Months | 3337.4 | 1, 112 | 0.411 | 0.523 |
| *Pocillopora damicornis* | DHW | 8856 | 1, 542.59 | 15.932 | **<0.001** |
|  | Months | 68480 | 1, 542.89 | 123.204 | **<0.001** |
| *Pocillopora meandrina* | DHW | 5583 | 1, 1930.83 | 1.579 | 0.209 |
|  | Months | 362572 | 1, 1931.50 | 102.569 | **<0.001** |
|  | Habitat | 47729 | 1, 182.13 | 13.502 | **<0.001** |
|  | Months * Habitat | 60392 | 1, 1931.92 | 17.085 | **<0.001** |
| *Stylophora pistillata* | DHW | 6898.1 | 1, 97 | 0.547 | 0.461 |
|  | Months | 12560.6 | 1, 97 | 0.996 | 0.321 |

Statistical output from a Type-I (or Type-III for interactions) analysis of variance (ANOVA) for the effects of Degree Heating Weeks (DHW), month, and/or habitat on live planar area of individual coral colonies, by species. Bold indicates statistical significance (𝛼 = 0.05).
