# Supplementary material for: Inter- and intraspecific responses of coral colonies to thermal anomalies on Palmyra Atoll, central Pacific
Source: PLoS One. 2024 Nov 25;19(11):e0312409. doi: 10.1371/journal.pone.0312409 (PMC11588205; doi:10.1371/journal.pone.0312409)
Supplement: S5 Table — Statistical output from a Type-I (or Type-III for interactions) analysis of variance (ANOVA) for the effects of Degree Heating Weeks (DHW), month, and/or habitat on percent discoloration of individual coral colonies, by species. Bold indicates statistical significance (α = 0.05). (DOCX) [file pone.0312409.s011.docx]

**S5 Table.** **ANOVA results for percent discoloration of coral colonies by species.**

| Species | Source | SumSq | Df  (num, den) | F value | Pr(>F) |
| --- | --- | --- | --- | --- | --- |
| *Astrea curta* | DHW | 950.08 | 1, 149.49 | 7.105 | **0.009** |
|  | Months | 137.90 | 1, 159.03 | 1.031 | 0.311 |
| *Astreopora myriophthalma* | DHW | 328.95 | 1, 60.647 | 1.699 | 0.197 |
|  | Months | 1417.84 | 1, 65.832 | 7.323 | **0.009** |
| *Goniastrea stelligera* | DHW | 1224.37 | 1, 405.98 | 5.567 | **0.019** |
|  | Months | 373.34 | 1, 444.78 | 1.698 | 0.193 |
|  | Habitat | 1975.87 | 1, 66.39 | 8.984 | **0.004** |
|  | Months * Habitat | 606.36 | 1, 446.38 | 2.757 | 0.098 |
| *Hydnophora microconos* | DHW | 1962.53 | 1, 92.655 | 7.431 | **0.008** |
|  | Months | 119.35 | 1, 93.771 | 0.452 | 0.503 |
| *Pavona chiriquiensis* | DHW | 546.39 | 1, 328.51 | 4.8229 | **0.029** |
|  | Months | 1993.47 | 1, 345.10 | 17.596 | **<0.001** |
| *Pavona duerdeni* | DHW | 24.878 | 1, 86.325 | 0.132 | 0.717 |
|  | Months | 227.437 | 1, 90.149 | 1.208 | 0.275 |
| *Pocillopora damicornis* | DHW | 454.35 | 1, 191.20 | 5.589 | **0.019** |
|  | Months | 83.30 | 1, 205.22 | 1.025 | 0.313 |
| *Pocillopora meandrina* | DHW | 2310.75 | 1, 943.11 | 20.973 | **<0.001** |
|  | Months | 9.51 | 1, 1073.71 | 0.0863 | 0.769 |
|  | Habitat | 2639.75 | 1, 205.88 | 23.959 | **<0.001** |
|  | Months * Habitat | 5.85 | 1, 1073.17 | 0.053 | 0.818 |
| *Stylophora pistillata* | DHW | 4727.7 | 1, 66.828 | 70.608 | **<0.001** |
|  | Months | 91.2 | 1, 70.041 | 1.363 | 0.247 |

Statistical output from a Type-I (or Type-III for interactions) analysis of variance (ANOVA) for the effects of Degree Heating Weeks (DHW), month, and/or habitat on percent discoloration of individual coral colonies, by species. Bold indicates statistical significance (𝛼 = 0.05).
